# Far‐from‐equilibrium thermodynamics of the human uterus: A self‐organized dissipative structure

**DOI:** 10.14814/phy2.70483

**Published:** 2025-10-02

**Authors:** Yves Lecarpentier, Victor Claes, Xénophon Krokidis, Christophe Locher, Jean‐Louis Hébert, Olivier Schussler, Francine Michel

**Affiliations:** ^1^ Centre de Recherche Clinique Grand Hôpital de l'Est Francilien Meaux France; ^2^ Department of Pharmaceutical Sciences University of Antwerp Wilrijk Belgium; ^3^ Service d'Hépato‐Gastro‐Entérologie et d'Oncologie Digestive Grand Hôpital de l'Est Francilien Meaux France; ^4^ Institut de Cardiologie, Hôpital de la Pitié‐Salpêtrière Assistance Publique‐Hôpitaux de Paris Paris France; ^5^ Département de Chirurgie Thoracique, Hôpital Cochin, Hôpitaux Universitaires Paris Centre Paris‐Descartes Université, Assistance Publique‐Hôpitaux de Paris Paris France; ^6^ Service de Gynécologie‐Obstétrique Grand Hôpital de l'Est Francilien Meaux France

**Keywords:** bifurcation, dissipative structure, far‐from‐equilibrium, self‐organization, thermodynamics, uterus

## Abstract

The aim of this study was to know how the human uterine muscle behaves from a thermodynamic point of view in pregnant and non‐pregnant states. According to far‐from‐equilibrium thermodynamics, an open system is far‐from‐equilibrium when its thermodynamic force varies non‐linearly with its thermodynamic flow. These two quantities can be calculated on muscles and therefore on the uterine muscle, using the Huxley formalism. The thermodynamic flow was greater in non‐pregnant compared to pregnant uterine muscle, while the thermodynamic force was equal in both cases. There was a linear relationship between these two quantities in pregnant uterine muscle, whereas a non‐linear relationship characterized the non‐pregnant state. Thus, the non‐pregnant uterine muscle worked far‐from‐equilibrium. The product of thermodynamic force and thermodynamic flow was the energy production rate (EPR). EPR was much greater in pregnant uterine muscle. The derivative with respect to time of EPR was called excess entropy production (EEP). EEP was positive in the pregnant state and negative in the non‐pregnant state. A first‐order nonlinear differential equation with one parameter demonstrated a bifurcation. These results showed that the non‐pregnant uterine muscle was a self‐organized far‐from‐equilibrium dissipative structure.

## 1 INTRODUCTION

How does the uterus work in women in periods as different as pregnancy or non‐pregnancy? During pregnancy, the uterus adapts to a protective role, while acquiring a function of expulsion of the fetus necessary for childbirth. The uterus undergoes a progressive and long evolution until its final term, which is childbirth. At the end of pregnancy, the uterus prepares for childbirth with the occurrence of contractions that are more and more frequent, organized, and strong.

Muscles are open systems and consequently are not in thermodynamic equilibrium. Like all living systems, they operate out of equilibrium. They are maintained in a non‐equilibrium state by a constant flow of matter and energy, either in a near‐equilibrium stationary linear stationary regime or far‐from‐equilibrium (Lecarpentier et al., 2005; Nicolis & Prigogine, 1979). Some muscles have been reported to work in a stationary linear regime when thermodynamic force linearly varies with thermodynamic flow (Kondepudi, 1999). On the contrary, others operate far‐from‐equilibrium as soon as the thermodynamic flow ceases to be linearly related to the thermodynamic force. Statistical entropy measures the organizational status of a system and depicts the evolution of this system at a given time. It does not drive, cause, or affect the evolution of the system. This is assigned to the entropy production rate, which quantifies irreversible chemical processes in a system (Kondepudi, 1999). In muscle tissues, the huge number of myosin involved in contractile processes provides the necessary ground for applying statistical mechanics. This science establishes an excellent link between microscopic and macroscopic thermodynamic properties in both physical and biological systems (Atkins, 1990). Statistical mechanics makes it possible to determine the entropy production rate quantifying irreversible chemical processes. For applying statistical mechanics in living muscles, a description of the behavior of myosin molecular motors is needed and is provided by A. Huxley's equations (Huxley, 1957). They establish a relationship between myosin kinetics and the mechanics of the whole muscle. Furthermore, they make it possible to determine the probability of steps of the actomyosin cycle (Lymn & Taylor, 1971). This is the cornerstone for using statistical mechanics as a tool for the mathematical solution of A. Huxley's phenomenological formalism.

From a thermodynamic viewpoint, we do not know whether or not the pregnant woman's uterus changes its behavior relative to the thermodynamic equilibrium. Is the uterus close or far‐from‐equilibrium? Does it change its thermodynamic mode depending on whether the woman is pregnant or not pregnant? We will see that after childbirth, the uterus moves far away from equilibrium, undergoes a process of self‐organization, and becomes a dissipative structure (Glansdorff et al., 1974; Kondepudi, 1999; Nicolis & Prigogine, 1979; Prigogine et al., 1974). Conversely, in pregnant women and at the time of delivery, the uterus becomes close to equilibrium, behaves like a classic smooth muscle driven by complex hormonal changes, and loses its self‐organized character and dissipative structure. At the time of delivery, the progesterone that maintained the quiescent myometrium throughout the pregnancy plays a major role in the fall in uterine contractility through its withdrawal (Shynlova et al., 2023). Dissipative structures are non‐equilibrium systems that evolve to an ordered state as a result of slight fluctuations (Prigogine, 1986; Prigogine et al., 1974). They are open systems appearing far‐from‐equilibrium and exchanging energy and matter with the external environment. Because entropy and free‐energy dissipating irreversible processes generate and maintain these structures, these have been called dissipative structures by I. Prigogine. They are numerous in the field of physics and chemistry. They include the Belousov‐Zhabotinsky reaction, which is a basically a catalytic oxidation of an organic compound such as malonic acid (Zhabotinsky, 1991). They include Bénard‐Taylor convection, turbulence, hurricanes, vortices (Kondepudi, 1999), tribology (Nosonovsky, 2010) and fatigue (Naderi, 2020). In biology, there are Turing structures (Turing, 1990), biomolecular handedness, chirality, enantiomers, and racemization (Frank, 1953), allosteric models (Goldbeter & Lefever, 1972) and biological rhythms (Goldbeter et al., 2012). However, they are relatively rarely described in the field of medicine, in cancer (Lefever & Garay, 1977), bipolar disorder (Goldbeter, 2013), tribology of the heart (Lecarpentier et al., 2022) and myocardial anoxia (Lecarpentier et al., 2024).

## 2 METHODS

### 2.1 Ethics Statement

This study was a human tissue study. Human biological samples were obtained from women who underwent either a caesarean or a hysterectomy outside of pregnancy in the gynecology‐obstetric department of the Grand Hôpital de l'Est Francilien in Meaux, France. The study was conducted in accordance with the Declaration of Helsinki. The Institutional Review Board‐The Comité des Personnes (CPP Ile de France XI, Afssaps: 2008‐A0039‐46) CPP: 08015)‐gave a favorable opinion. All patients gave oral and written informed consent with the Local Ethical Committee approval.

### 2.2 Mechanics of human uterus samples from caesarean and hysterectomy

For women who had a caesarean (*n* = 23), a small fragment of < 5 g was taken from the intermediate segment. For hysterectomized women (*n* = 20), a small fragment of the uterus was obtained, outside of menstrual periods. Uterine samples were collected from healthy areas of the uterus, that is, free from macroscopic abnormalities. The age of the women ranged from 22 to 43 years for caesarean (32 ± 6 years) and 24–72 years for hysterectomy (49 ± 6 years). Body weight and height did not differ in the two groups of women. Maximum blood pressure was slightly higher in hysterectomy than in caesarean, but in neither case was there hypertension. Similarly, there was no diabetes or renal failure in either type of woman (Table 1). Table 1 shows the main clinical signs of the women.

**TABLE 1:** Clinical signs of women.

Mean ± SD	Caesarean	Hysterectomy	*p*
Age (years)	32 ± 6	49 ± 6	*p* < 0.001
Body weight (kg)	66 ± 12	69 ± 10	NS
Height (m)	1.6 ± 0.1	1.6 ± 0.1	NS
Systolic pressure (mmHg)	110 ± 7	118 ± 8	*p* < 0.01
Diastolic pressure (mmHg)	72 ± 9	75 ± 8	NS
Glycemia (mmol/L)	4.8 ± 0.2	5.6 ± 0.8	NS
Creatinemia (μmol/L)	50 ± 7	66 ± 12	*p* < 0.05

In caesarean, there were no cases of gestational hypertension. In both cases, the samples were collected and transported in a physiological saline solution to the mechanical laboratory within 5 min (Krebs–Henseleit solution (in mM): 118 NaCl, 4.7 KCl, 1.2 MgSO_4_.7 H_2_O, 1.1 KH_2_PO_4_, 24 NaHCO_3_, 2.5 CaCl_2_.6H_2_O, and 4.5 D‐glucose). Arriving at the mechanical laboratory, the fragments were cut and quickly installed on a force and length transducer. The myometrial strips were excised from the outer longitudinal layer and dissected free of serosa. Myometrial fragments were suspended in a bath chamber containing a Krebs–Henseleit solution and maintained at 29°C. The solution was bubbled with a gas mixture (95% O_2_‐5% CO_2_). This made it possible to maintain a pH at 7.4. Experimental samples were allowed to equilibrate during 1 h with a resting force of 2 mN. At the end of the equilibration period, in 90% of uterus strips, spontaneous and regular contractions generally occurred. When spontaneous contractions did not develop, muscle samples were discarded. The electromagnetic apparatus has been previously described (Lecarpentier et al., 1998).

Experiments were carried out at Lo, which was the resting initial length that corresponded to the apex of the Frank‐Starling curve (see Appendix A). Maximum shortening velocity (*V*_max_ in Lo.s^−1^) was calculated by means of the zero‐load clamp technique (Brutsaert & Claes, 1974). Maximum isometric tension (TT in mN/mm^2^) was measured from the fully isometric tension. The isotonic tension (iT)‐velocity (V) relationship was constructed from 6 to 8 afterloaded curves from zero‐load up to isometric tension. This relationship was fitted to the Hill hyperbola:

(1) $$\left(V+b\right)\;\left(\mathrm{iT}+a\right)=b\;\left(\mathrm{TT}+a\right)$$

‐a and ‐b were the asymptotes of the hyperbolic curve (Csapo, 1954; Hill, 1938). The G curvature of the Hill equation was *V*_max_/b = TT/a.

### 2.3 A. Huxley's formalism

The formalism of A. Huxley was treated in the Appendix A (Huxley, 1957). It allows to calculate the molecular properties of the myosin crossbridges (CB): po was the individual CB force; *f*_1_ was the attachment rate constant and *g*_1_ and *g*_2_ were the detachment rate constants; kcat was the catalytic constant; and myosin Ca^2+^ ATPase activity was equal to the product of kcat and myosin content. According to A. Huxley's theory, only one ATP molecule was split per CB cycle. The molecular step size h was the translocation distance of the actin filament per ATP hydrolysis, produced by the swing of the myosin head. Time stroke (ts) was the duration of the CB translocation (h) (see Appendix A).

### 2.4 Thermodynamic approach: Near or far‐from‐equilibrium

Uterine muscles are living open systems exchanging energy and matter with the outside world. The first step was to determine whether the uterus was operating close to or far from equilibrium. It operated close to equilibrium when the thermodynamic force varied linearly with the thermodynamic flow. Conversely, it operated far from equilibrium when the thermodynamic force breached its linearity with the thermodynamic flow. Thermodynamic force (Eo/T) was a function of the myosin concentration (Eo = *f* (MC) = 4.24 (MC^2^) divided by the Kelvin temperature T) (see Appendix A). Thermodynamic flow was the sliding velocity of myosin CB (v0).

### 2.5 Self‐organization and criteria of stability or instability

The occurrence of self‐organization imposes that the system was open and far‐from‐ equilibrium. The entropy production rate (EPR) was obtained by the following expression:

(2) $$y=\mathrm{EPR}=\delta^{2}S=\left(1/T\right)\times \mathrm{Eo}\times \text{v0}$$

where S is the entropy of the system and T the temperature. The myosin ATPase is a special enzyme due to the fact that the power stroke and the hydrolysis chemical step occur at different stages of the ATP‐actin‐myosin cycle (Huxley, 1957; Rayment et al., 1993; Spudich, 1994). The seminal work of A. Huxley (Huxley, 1957) allows us to calculate both the thermodynamic flow (v0) and the thermodynamic force Eo/T.

The stability condition for the thermodynamic system was given by the equation:

(3) $$y^{\prime }=\frac{\partial \delta^{2}S}{\mathrm{\partial t}}=\left(1/T\right)\times \mathrm{\delta Eo}\times \text{δv0}>0$$

where $\frac{\partial \delta^{2}S}{\mathrm{\partial t}}$ was the excess entropy production (EEP).

The system became unstable when equation 3 became negative.

(1/T) δEo and δv0 were respectively the deviations of the thermodynamic force (Eo/T) and the thermodynamic flow (v0) from the stationary state. The system remained stable as long as the EEP was positive. When the deviation from the stationary state went beyond a certain critical value, the system reached an instability threshold. EEP could change its sign at a given value and then became negative and unstable. A transition to a self‐organized solution was able to occur. It was difficult to define a single criterion for the occurrence of self‐organization. The criterion based on the loss of thermodynamic stability (i.e., EEP <0) defined well the occurrence of self‐organization (Fox‐Rabinovich et al., 2018; Gershman et al., 2023; Glansdorff et al., 1974; Nicolis, 1977; Nicolis & Prigogine, 1979; Nosonovsky, 2010). The necessary condition for the occurrence of self‐organization was EEP negative. This was a pre‐requisite for self‐organization.

### 2.6 Determination of a one‐dimensional non‐linear equation with one parameter ψ

We applied then a polynomial regression between *y*′ and *y*, and obtained a relationship between y' and *y*. We found a one‐dimensional non‐linear differential equation. A term (G curvature) of this equation was related to *y*′^2^ which was introduced in the differential equation. A one‐dimensional non‐linear differential equation with one parameter (ψ) was then obtained. We studied the phase diagram *y*′ = *f* (*y*, ψ) and the bifurcation diagram *y* = *f* (ψ). On the phase diagram, *y*′ = *f* (*y*, ψ) shows a non‐hyperbolic fixed point (y*) because λ = *f*′(y*) was equal to 0. A bifurcation occurred at the non‐hyperbolic fixed point.

In a thermodynamic system, dissipative structures occurred under several conditions. The system must be open, exchanging energy, matter and information with the exterior. The system must operate far‐from‐equilibrium and under a non‐linear regime. This was characterized by the non‐linearity between the thermodynamic force and the thermodynamic flow. The self‐organized system imposed a negative EEP. The system must be subjected to slight fluctuations. Dissipative structures disappeared if the exchange of energy and matter between the system and its environment ceased.

### 2.7 Statistical analysis

Data were expressed as means ± standard deviations. ANOVA was carried out using the Statview software to determine statistically significant differences. A *p* value <0.05 was considered statistically significant.

## 3 RESULTS

### 3.1 Mechanical parameters of uterus and CB kinetics during caesarean and hysterectomy

**FIGURE 1:**
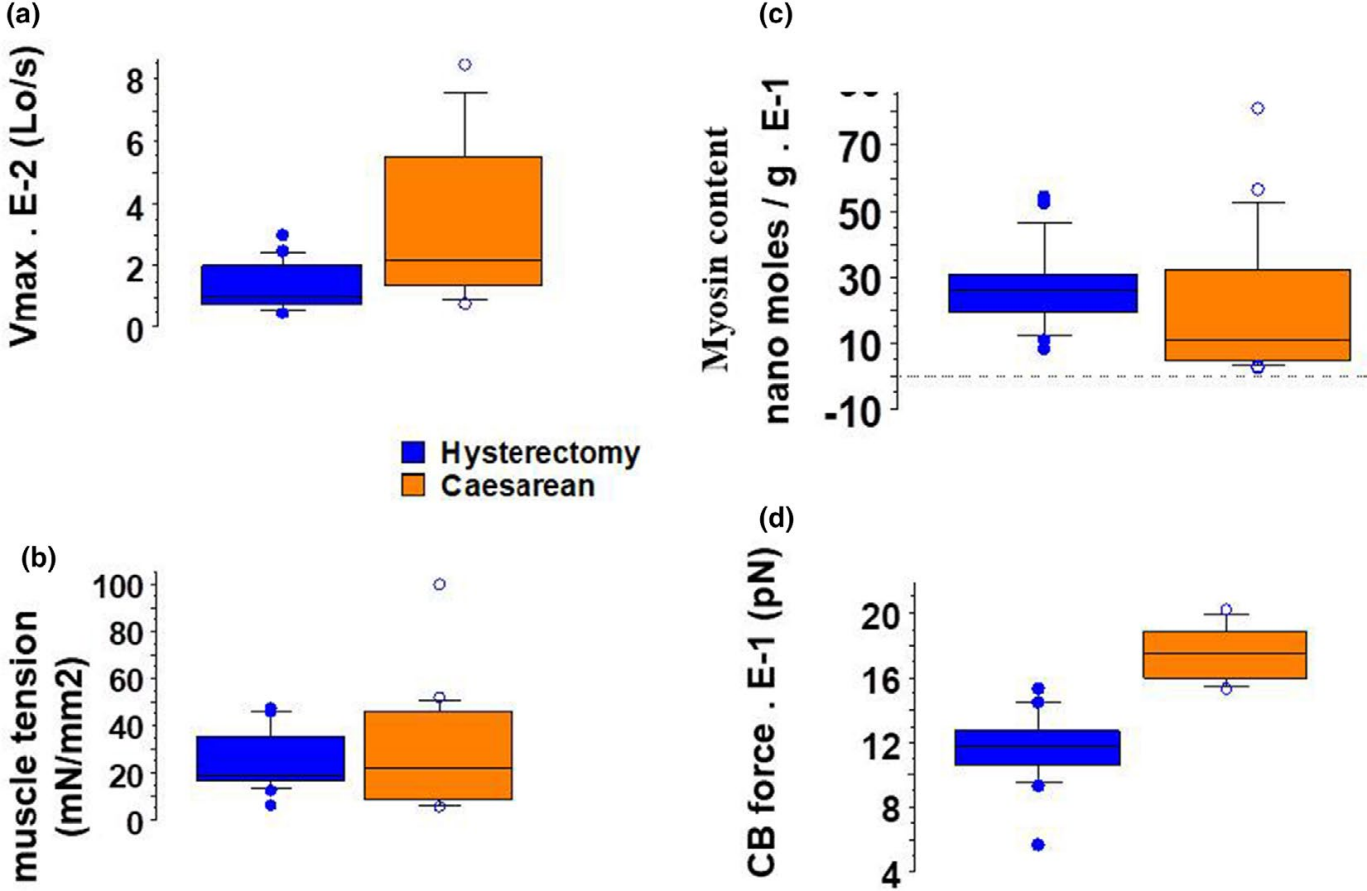
Mechanical parameters of uterus and CB kinetics. Box plot; In caesarean, *n* = 23; in hysterectomy, *n* = 20. (a) *V*_max_ (in Lo/s); (b) Muscle tension (in mN/mm^2^); (c) Nano moles/g of tissue; (d) CB force (in pN).

**FIGURE 2:**
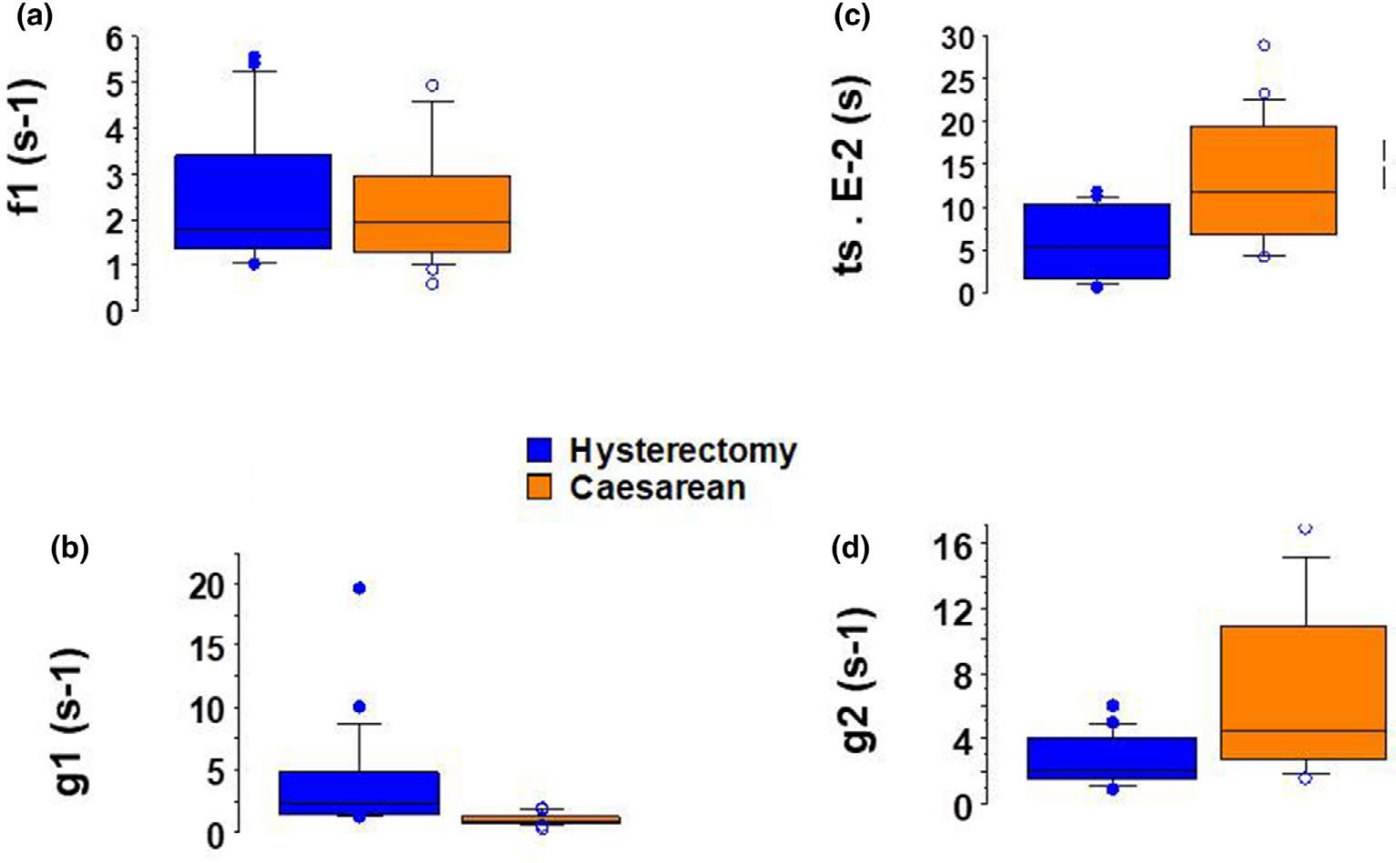
Molecular myosin CB characteristics. Box plot; In caesarean, *n* = 23; in hysterectomy, *n* = 20. (a) Rate constant for CB attachment (*f*1); (b) Rate constant for CB detachment (*g*1); (c) Time stroke (ts); (d) Rate constant for CB detachment (*g*2).

**FIGURE 3:**
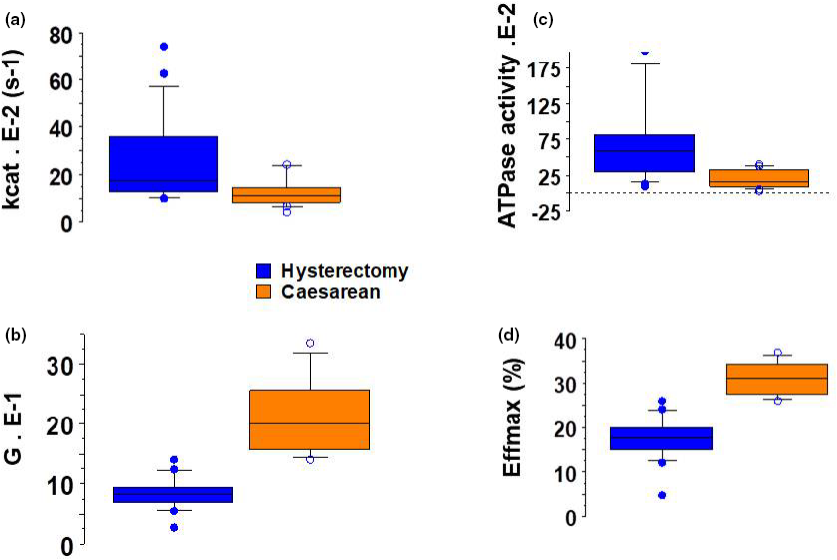
Biochemical characteristics of CB. Box plot; In caesarean, *n* = 23; in hysterectomy, *n* = 20. (a) kcat, in s^−1^; (b) G curvature (without dimension); (c) Myosin ATPase activity in nmol/g/s; (d) Effmax in %.

**FIGURE 4:**
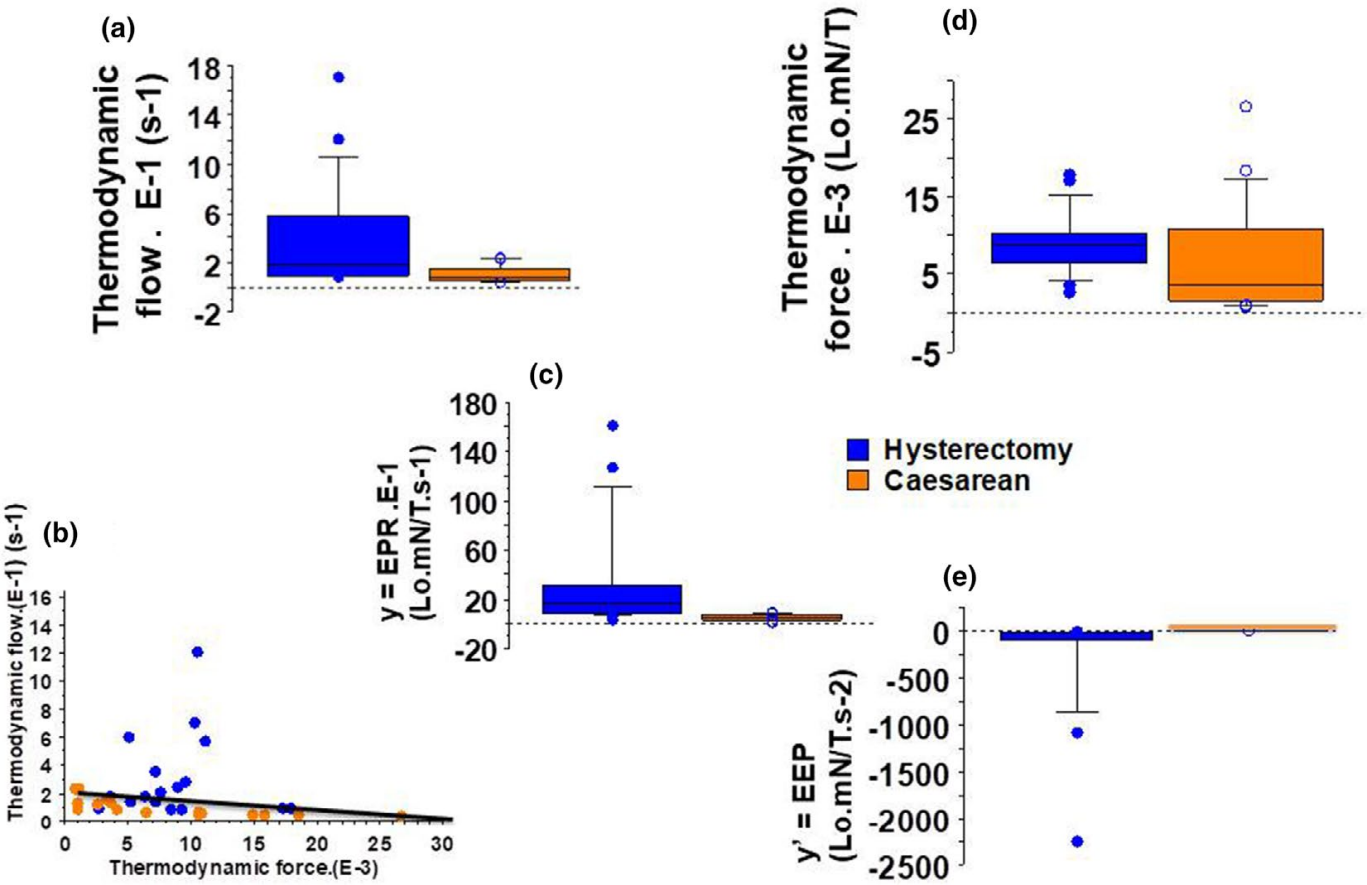
Thermodynamics characteristics in Hysterectomy and Caesarean. Box plot; In caesarean, *n* = 23; in hysterectomy, *n* = 20. (a) Thermodynamic flow (in s^−1^); (b) Thermodynamic flow (in mN) versus thermodynamic force relationship (in Lo.mN/T): (c) Energy production rate (*y* = EPR, in Lo.mN/T.s^−1^); (d) Thermodynamic force; (e) Excess entropy production (*y*′ = EEP in Lo.mN/T.s^−2^).

Box plots are a standardized, graphical way of summarizing the distribution of two sets of uterus data. They enable the display of five different values–the minimum, first quartile, median, third quartile, and maximum–in a single box shape for each group (from Figures 1, 2, 3, 4). The single points on the diagram show the outliers.

The maximum shortening velocity was much higher in Caesarean than in Hysterectomy (Figure 1a, Table 2). The same was observed for the force developed by an individual myosin CB (Figure 1d, Table 2). The total tension was slightly greater in Caesarean than in Hysterectomy but did not reach significance (Figure 1b, Table 2). The number of moles per g of tissue did not differ significantly in the two groups (Figure 1c, Table 2).

**TABLE 2:** Mean values ± standard deviations of uterus mechanical parameters.

	Hysterectomy	Caesarean	*p*
*V*_max_ (Lo/s)	0.013 ± 0.008	0.034 ± 0.026	0.002
Tension (mN)	20.5 ± 12.5	28.3 ± 23.9	NS
Myosin content (nano mole/g)	2.7 ± 1.2	2.2 ± 2.2	NS
CB force (pN)	1.2 ± 0.2	1.8 ± 0.2	0.0001
f1 (s‐1)	2.6 ± 1.5	2.3 ± 1.3	NS
g1 (s‐1)	3.9 ± 4.5	1.0 ± 0.5	0.01
g2 (s‐1)	2.6 ± 1.5	6.9 ± 5.1	0.001
ts (s)	0.06 ± 0.04	0.13 ± 0.07	0.001
kcat (s‐1)	0.26 ± 0.18	0.12 ± 0.05	0.005
ATPase activity (nano mole/g/s)	0.72 ± 0.60	0.18 ± 0.13	0.001
G curvature	0.85 ± 0.27	2.14 ± 0.66	0.001
Effmax (%)	17.7 ± 5.0	31.1 ± 3.7	0.001
Thermo flow. E‐1 (s‐1)	3.9 ± 4.4	1.1 ± 0.6	0.005
Thermo force. E‐3 (Lo.mN/T)	8.7 ± 3.9	7.3 ± 7.3	NS
*y* = EPR. E‐1 (Lo.mN/T.s‐1)	35.0 ± 44.3	4.6 ± 2.4	0.005
*y*′ = EEP (Lo.mN/T.s‐2)	−237 ± 564	0.9 ± 0.8	0.05

The CB attachment rate constant (*f*1) did not differ in the two groups (Figure 2a, Table 2). The CB detachment rate constant (*g*1) was higher during Hysterectomy than during Caesarean (Figure 2b, Table 2) and the opposite was observed for the CB detachment rate constant (g2) (Figure 2d, Table 2). The time stroke (ts) was greater during Caesarean than in Hysterectomy (Figure 2c, Table 2).

Kcat (Figure 3a, Table 2) and myosin ATPase activity (Figure 3c, Table 2) were greater during Hysterectomy than during Caesarean. The curvature of the hyperbola (G) was greater during Caesarean than during Hysterectomy (Figure 3b, Table 2). The same was true for the maximum efficiency (Effmax) (Figure 3d, Table 2).

### 3.2 Thermodynamic statistics: Was the uterus a near‐equilibrium or a far‐from‐equilibrium system?

The myofilament sliding velocity (v0) was the thermodynamic flow. Eo divided by the temperature (T) was the thermodynamic force. The thermodynamic flow was greater in Hysterectomy than in Caesarean (Figure 4a, Table 2). The thermodynamic force was of the same order of magnitude in the two groups (Figure 4d, Table 2).

In Caesarean, the thermodynamic flow was linear with the thermodynamic force (Figure 4b). Thus, the uterus was near‐equilibrium in this group. On the contrary, in Hysterectomy, there was no linear relationship between the thermodynamic flow and thermodynamic force (Figure 4b) and the uterus was far‐from‐equilibrium. Thus, in Caesarean, the uterus was a near‐equilibrium system. However, in Hysterectomy, when it became outside the period of childbirth, the uterus was a far‐from‐equilibrium and self‐organized system.

### 3.3 Energy production rate (EPR)

**FIGURE 5:**
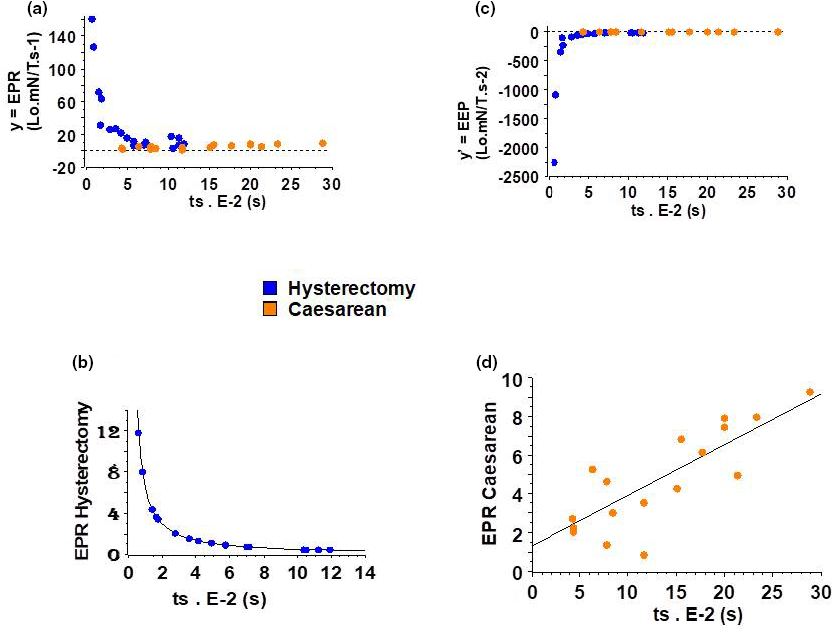
Thermodynamic characteristics in Hysterectomy and Caesarean. (a) EPR versus time stroke relationship; (b) EPR hysterectomy versus time stroke relationship; (c) EEP versus time stroke relationship; (d) EPR caesarean versus time stroke relationship.

Energy production rate (*y* = EPR) was the product of thermodynamic flow and thermodynamic force (Figure 4c) and was greater in Hysterectomy than in Caesarean. In Figure 5a, *y* = EPR was shown as a function of ts during which the CB operated its stroke (ts: time stroke). In Caesarean, *y* was slightly increasing with ts, and fitted with a first polynomial degree (Figure 5d). The derivative of *y* according to ts was positive and linearly increasing with ts. In Hysterectomy, *y* = EPR dramatically decreased with ts and fitted with a power function *y*. (E‐3) = 0.99 ts^−0.69^ (Figure 5b). The derivative of *y* was negative and tended towards zero.

### 3.4 Excess entropy production (EEP)

The derivative of *y* according to ts was the Excess Entropy Production (*y*′ = EEP) (Figures 4e and 5c). It was positive in Caesarean. In Hysterectomy, it was negative and became zero at ts = 0.12 s. In Hysterectomy, the negativity of EEP shows that uterus was in the domain of self‐organized structures.

### 3.5 Determination of a one‐dimensional non‐linear differential equation

Let *y* = EPR = v0 × Eo/T be expressed as a function of *y*′ according to a second polynomial degree:

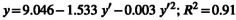




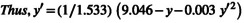

$$y^{\prime }=\left(0.65\right)\;\left(9.046-y-0.003\;{y^{\prime }}^{2}\right)$$

This was a one‐dimensional non‐linear differential equation.

### 3.6 Determination of the parameter ψ of the one‐dimensional non‐linear equation

*y*′^2^ could be expressed as a power function of the G curvature:

$${y^{\prime }}^{2}=6.89\;\left(G-4.82\right)$$

Thus, the one‐dimensional non‐linear equation with one parameter ψ, function of G, could be written as follows:

$$y^{\prime }=\left(0.65\right)\;\left(9.046-y-0.02G-4.82\right)$$

### 3.7 Phase diagram: *y*’ = *f* (*y*, ψ)

**FIGURE 6:**
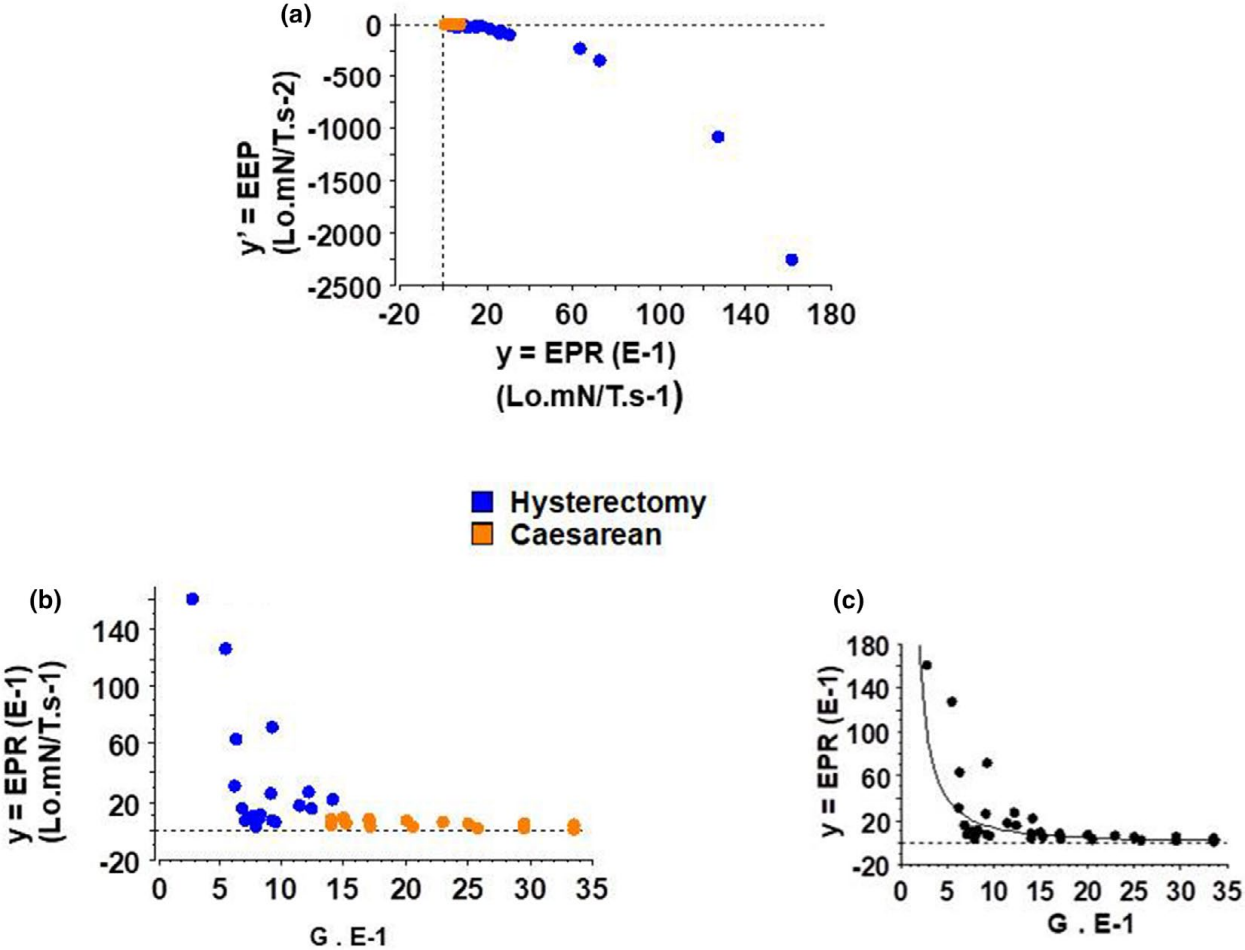
Entropy production rate (EPR) and excess entropy production (EEP). (a) Phase diagram *y*′ = *f* (*y*, ψ); (b) EPR versus G relationship, bifurcation diagram: *y* = *f* (ψ); (c) EPR versus G fit; bifurcation diagram *y* = *f* (ψ) showing the change of direction at the transition hysterectomy‐caesarean (Figure 6b,c).

The equation *y*′ = *f* (*y*, ψ) represented the phase diagram (Figure 6a).

### 3.8 Bifurcation diagram *y* = *f* (ψ)

The bifurcation diagram *y* = *f* (ψ) presented a dramatic change of direction at the transition Hysterectomy‐Caesarean (Figure 6B). The bifurcation occurred at about *G* = 1.

When the parameter of the equation *f* (ψ) = *f* (G) decreased from 3.5 to 1, *y* = EPR decreased but the slope was very slight. This was the domain of Caesarean. At G = 1, there was a sudden change in the EPR direction and the slope dramatically and suddenly increased, thus marking a bifurcation. This was the domain of Hysterectomy. This bifurcation occurred at the fixed point of the non‐linear differential equation of the far‐from‐equilibrium system. At the bifurcation, the value of the parameter G was about 1.

### 3.9 Stability analysis

The local stability of the one‐dimensional non‐linear differential equation *y*′ = *f*(*y*, ψ) was determined by examining the first derivative of the function *y* = EPR evaluated at the fixed point y*. Let λ be equal to *f*′(y*). λ was equal to 0. Thus y* was a non‐hyperbolic fixed point. The slope of the function *y*′ = *f* (*y*, ψ) at the fixed point y* determined the stability of the differential equation. The non‐hyperbolic fixed point was half‐stable (unstable) because trajectories converged at the fixed point on one side but diverged from the fixed point on the other side. In this hybrid case, in Hysterectomy the fixed point was attracting with a negative slope of *y*′ = *f* (*y*, ψ). Conversely in Caesarean, the fixed point was repelling with a positive slope of *y*′ = *f* (*y*, ψ).

The uterus lost its thermodynamic stability at the fixed point. EEP became negative. This was a prerequisite for self‐organization to begin. A dissipative structure appeared in Hysterectomy and outside of pregnancy under the following conditions: (i) the uterus was an open system, which is the case for all living systems; it exchanged energy and matter with its environment; (ii) in Hysterectomy, it operated far‐from‐equilibrium and under a non‐linear regime, as attested by the non‐linearity between the thermodynamic flow and the thermodynamic force; (iii) it was subjected to slight fluctuations during this period. This modified the molecular properties of the myosin crossbridges and particularly the CB detachment rate constants. The unitary myosin CB force and the G curvature of the T‐V relationship depended on them and accounted for the thermodynamic results.

## 4 DISCUSSION

### 4.1 Conditions of formation of dissipative structures

Schrödinger in his book “What is life” (Schrödinger, 1967) establishes a link between biology, physics and chemistry, and defines order, disorder and entropy. In the life time, Prigogine's theory states that entropy production rate tends to decrease in open biological systems, since an orderly structure is need to be maintained. Dissipative structures have the ability of self‐organization into complex structures. They maintain in space and time, and tend to conserve a stationary state far‐from‐equilibrium with a low entropy (Glansdorff et al., 1974; Kondepudi, 1999; Nicolis & Prigogine, 1979; Prigogine et al., 1974).

### 4.2 Experimental conditions

The study was carried out on isolated uterine fragments and not on an entire uterus in situ. The muscles were studied under identical conditions: pH and composition of the Krebs–Henseleit solution. The uterine fragments were of the same shape and size in both groups. The temperature was the same. This decreases problems inherent in the complex spatial geometry of the uterus and in the fact that on the entire organ there are several layers (endometrium, myometrium). Caution should be exercised when interpreting the results, as the conditions under which the uterus evolved during caesarean and hysterectomy were so different. Metabolic, mechanical, and energetic conditions differed, and this must be taken into account. The T‐V curve is well known to have a thermodynamic significance: the higher G, the more economical the muscular system. The curvature G of the Hill hyperbolic relationship was related to the myosin isoform type. The higher the G, the slower the myosin isoform (Schwartz et al., 1981). G was also related to thermodynamic parameters: the higher the G, the more economical the muscle (Alpert & Mulieri, 1982).

For applying statistical mechanics in living muscles, a description of the behavior of myosin molecular motors was needed and was provided by A. Huxley's equations (Huxley, 1957). Equations established a relationship between myosin kinetics and the mechanics of the whole muscle. This was the cornerstone for using statistical mechanics as a tool for mathematical solution of A. Huxley's phenomenological formalism. A main reason led us to prefer A. Huxley's model. Huxley himself writes in his seminal article (Huxley, 1957): “It is natural to ask whether the mechanism proposed here for striated muscle could account also for the contraction of smooth muscle. On general grounds, it is to be expected that the mechanism is fundamentally the same in both types, so that it would be unsatisfactory to postulate for one type a mechanism that clearly cannot exist in the other”.

The theory applies equally well to sarcomeric skeletal muscles and to smooth muscles devoid of sarcomeres. Several authors have applied Huxley's formalism to different types of smooth muscles in swine carotid artery (Hai & Murphy, 1988), in bovine trachea (Fredberg et al., 1999), and in airway smooth muscle of humans, rats, and rabbits (Lecarpentier et al., 2002). The ultrastructure of smooth muscles differs from that of striated muscles. There is no *Z*‐line. The attachment of actin filaments to dense bodies is reminiscent of that found at *Z* lines observed in striated muscle (Fay et al., 1983; Kargacin et al., 1989). In smooth myosin, crossbridges along a rodlike filament with no central bare zone project in opposite directions and on opposite sides of the filament. Myosin heads, along an entire side, have the same polarity along the entire filament length (Xu et al., 1996). Both smooth and skeletal muscle myosins produce a similar power stroke of 10 to 11 nm when measured with optical tweezers (Finer et al., 1994; Guilford et al., 1997; Lauzon et al., 1998). In our study, a power stroke of 11 nm was chosen. Furthermore, a power stroke has been shown to be on the same order of magnitude in crystallography analysis of the myosin motor domain of smooth muscle (Dominguez et al., 1998). The CB unitary force was of the same order of magnitude as those previously measured by means of the laser trap in both smooth and skeletal muscles and in intact skeletal muscle (Finer et al., 1994; Molloy et al., 1995). The other main reason we chose A. Huxley's smooth muscle model is that it allows us to calculate the two main elements of thermodynamics that we need, namely the thermodynamic force and the thermodynamic flow.

The low value of myosin ATPase activity in caesarean was associated with low values of thermodynamic flow and EPR. This was accompanied by a higher value of the Hill hyperbola curvature (G) and muscle efficiency. This behavior was consistent with that of a muscle that must ensure the birth of the newborn.

The age of the women was complex to analyze because it was higher in hysterectomy than in caesarean. However, hysterectomy was performed in postmenopausal women as well as in women with normal reproductive activity. However, the EPR did not distinguish them and was always low.

### 4.3 Close to delivery, the uterus operated near equilibrium, and outside of pregnancy, the uterus became a far‐from‐equilibrium self‐organized dissipative structure

During pregnancy, cellular processes take place to prepare the uterus to gradually acquire a muscle function specific to childbirth. Hormonal systems, in particular progesterone, play a determining role (Shynlova et al., 2023). During pregnancy, the uterus is to be quiescent. It does not contract. A major transition appears in the uterus labor with a breakdown of the feto‐maternal immune tolerance, cervical dilatation, and rise of myometrium contractility (Shynlova et al., 2023). Progesterone inhibits contraction‐associated proteins (CAPs). Progesterone acts as a brake on myocyte contractility (Csapo, 1970), particularly on CAP production. The increase in CAPs marks the transition from the non‐working phase of the uterus to the working phase. Thus, the CAP production increases at the end of pregnancy with both the decrease in progesterone and the increase in the estrogenic environment. In particular, the increase in one of the CAPs, Connexin 43, participates in the creation of gap junctions between myocytes, thus creating an electrical coupling between them. Uterine myocytes contract in response to both stretching and electrical stimulation. Depolarization of one cell can also cause depolarization of adjacent cells because cells are electrically connected by junctions made of connexin 43 (Garfield et al., 1988; Sakai et al., 1992; Sims et al., 1982). Progesterone is also implied in menstruation. Outside of pregnancy, uterine contractions are involved in the expulsion of menstrual debris at the time of menses. If an egg is not fertilized during the cycle, the corpus luteum breaks down, which decreases progesterone levels. This means the uterine lining thins and breaks down, causing the beginning of the menstrual period. It would be interesting to study the thermodynamic status of the uterus during this period.

A hypothesis for self‐organization in the human uterus during labor has been hypothesized, but the far‐from‐equilibrium thermodynamic state of the uterus and the negative excess entropy production were considered (Banney et al., 2015). The essential difference between the uterus near childbirth and the uterus outside pregnancy was the formation of a self‐organized behavior during the genital life outside pregnancy. In pregnant women close to childbirth, it is expected that the uterus acquires a contractile function ensuring effective delivery, with regular and powerful contractions. In this state, the uterus operates near equilibrium with linearity between thermodynamic flow and force. However, during the rest of life, the uterus largely has lost its contractile function, moving far away from equilibrium, to the point of beginning a process of self‐organization, allowing it to function according to a new rhythm. It is coordinated by a complex hormonal progesterone status that induces stimulation to maintain this state. In pregnant women close to childbirth, a new state of pregnancy driven by another progesterone hormonal circuit breaches this cycle of self‐organized processes and evolves toward near‐equilibrium, as evidenced by the linearity between the thermodynamic flow and the thermodynamic force, and allows the uterus to function as a true smooth contractile muscle.

## 5 CONCLUSION

The far‐from‐equilibrium thermodynamic analysis of the uterine behavior has made it possible to highlight a radically different functioning depending on whether it was examined at the time of childbirth or outside of pregnancy. In pregnancy and at the time of delivery, the uterine muscle was thermodynamically close to equilibrium with linearity between the thermodynamic flow and the thermodynamic force. Outside of a pregnancy, the uterus moved far‐from‐equilibrium, with a breakdown in the linearity between the thermodynamic force and flow. The uterus thus acquired a behavior of self‐organization and dissipative structure, linked to slight fluctuations acting on kinetics of myosin.

## AUTHOR CONTRIBUTIONS

**Yves Lecarpentier**: Conceptualization. **Yves Lecarpentier**, **Victor Claes**, **Xénophon Krokidis**, **Jean‐Louis Hébert**, and **Olivier Schussler**: Methodology. **Yves Lecarpentier**, **Victor Claes**, **Christophe Locher**, and **Francine Michel**: Software. **Yves Lecarpentier**, **Xénophon Krokidis**, and **Francine Michel**: Validation. **Yves Lecarpentier** and **Olivier Schussler**: Formal analysis. **Yves Lecarpentier**, **Xénophon Krokidis**, **Jean‐Louis Hébert**, and **Olivier Schussler**: Investigation. **Christophe Locher** and **Francine Michel**: Resources. **Yves Lecarpentier** and **Xénophon Krokidis**: Data curation. **Yves Lecarpentier**, **Victor Claes**, **Xénophon Krokidis**, **Christophe Locher**, **Jean‐Louis Hébert**, **Olivier Schussler**, and **Francine Michel**: Writing—original draft preparation. **Yves Lecarpentier** and **Xénophon Krokidis**: Writing—review and editing. **Yves Lecarpentier** and **Xénophon Krokidis:** visualization. **Yves Lecarpentier** and **Christophe Locher:** Supervision. **Christophe Locher** and **Francine Michel**: Project administration. **Yves Lecarpentier** and **Christophe Locher**: Funding acquisition.

## FUNDING INFORMATION

This research received no external funding.

## CONFLICT OF INTEREST STATEMENT

The authors declare no competing interests.

## ETHICS STATEMENT

This study is a human tissue study. Human biological samples were obtained from women who underwent either a caesarean or a hysterectomy in the gynecology‐obstetric department of the Grand Hôpital de l'Est Francilien in Meaux, France. The study was conducted in accordance with the Declaration of Helsinki. The Institutional Review Board “Comité des Personnes CPP Ile de France XI, Afssaps: 2008‐A0039‐46, CPP: 08015” gave a favorable opinion.

## INFORMED CONSENT STATEMENT

All patients gave oral and written informed consent with the Local Ethical Committee approval.

## Figures and Tables

**FIGURE A1:**
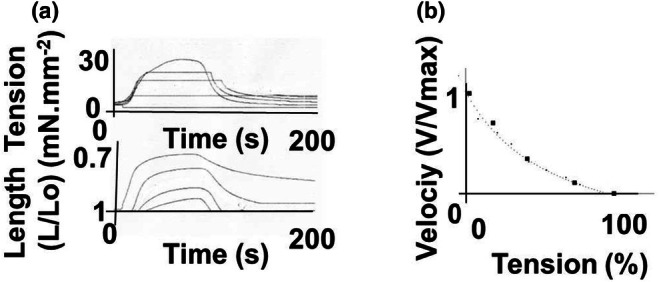
Schema of contractile properties. Contractile mechanics. Panel a: Tension and shortening length were represented as a function of time at five increasing load levels; Panel b: Hill hyperbolic Tension‐Velocity relationship with G curvature.

**FIGURE A2:**
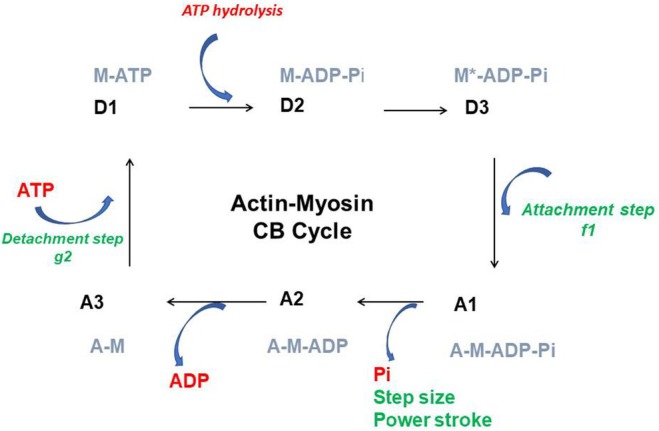
The actin‐myosin crossbridge cycle. The crossbridge cycle. The CB cycle was composed of six different conformational steps, that is, three detached steps (D1, D2, and D3) and three attached steps (A1, A2, and A3). The myosin molecular motor was the muscle myosin (M). Transition A3 → D1 was the ATP binding step that induced CB detachment after ATP binding with the actin (A)‐muscle myosin (M) complex (A‐M). The rate constant for detachment was g_2_; A‐M + ATP → A + M‐ATP. Transition D1 → D2 was the ATP hydrolysis: M+ ATP → M‐ADP‐Pi. Transition D2 → D3 was M‐ADP‐Pi → M*‐ADP‐Pi. D3 was the step with the highest probability. Transition D3 → A1 was the attachment state: The myosin head (M*‐ADP‐Pi) bonded with A and the rate constant for attachment was f_1_: M*‐ADP‐Pi + A → A‐M‐ADP‐Pi. Transition A1 → A2 was the power stroke which was triggered by the Pi release: A‐M‐ADP‐Pi → A‐M‐ADP + Pi. The power stroke was characterized by the generation of a unitary CB force. Transition A2 → A3 was the release of ADP: A‐M‐ADP → A‐M + ADP. The power stroke occurred with a tilt of the M head and produced a force of a few picoNewton and a displacement of a few nanometers.

## Data Availability

The data that support the findings of this study are available on request from the corresponding author.

## APPENDIX A

### A.1 CONTRACTILE PROPERTIES

The contractile properties of the uterine muscles were determined by measuring the tension and shortening of the uterine muscles as a function of time, at different load levels and from zero‐load clamp to isometric loading (Figure A1). The peak shortening velocity (V) was plotted for each load level (iT) (Figure A1). There was a hyperbolic relationship (V + b) (iT + a) = b (TT + a) between velocity and load level for both striated and smooth muscles (Lecarpentier et al., 1998). The curvature of the Hill hyperbola (G) was *V*_max_/b = TT/a and the asymptotes were ‐a and ‐b.

### A.2 THE ACTIN‐MYOSIN CROSSBRIDGE CYCLE

It was composed of six different steps. Values of the CB rate constants for attachement (f1) and detachment (g1 and g2) depended on the stage of the woman, that is, non pregnant or pregnant about to give birth. They determined all the CB molecular parameters (catalytic constant, myosin Ca^2+^ATPase activity and individual CB force) (Figure A2).

### A.3 HUXLEY'S FORMALISM

A. Huxley's physical formalism (Huxley, 1957) can be used for the mechanical study of striated sarcomeric muscles (skeletal and cardiac) and smooth muscles. It allows to calculate the mechanical properties of myosin at the molecular level. It required verifying that the tension‐velocity (P‐V) of each muscle specimen presented a hyperbolic profile. Values for the asymptotes ‐a and ‐b of the P‐V relationship were introduced into the Huxley equations. Briefly, equations expressed the isotonic tension ($P_{\mathrm{Hux}}$) and the rate of total energy release (E_Hux_′) as a function of muscle velocity (V):

(A1)
$$P_{\mathrm{Hux}}=N\frac{w}{1}\cdot \frac{f_{1}}{f_{1}+g_{1}}\left[1-\frac{V}{\phi }\left(1-e^{\frac{\phi }{V}}\right)\left(1+\frac{1}{2}{\left(\frac{f_{1}+g_{1}}{g_{2}}\right)}^{2}\frac{V}{\phi }\right)\right]$$

(A2)
$$E{'}_{\mathrm{Hux}}=\mathrm{Ne}\frac{h}{2l}\frac{f_{1}}{f_{1}+g_{1}}\left\{g_{1}+f_{1}\frac{V}{\phi }\left(1-e^{\frac{\phi }{V}}\right)\right\}$$

*f*_1_ was the peak value of the rate constant for myosin crossbridge (CB) attachment; and *g*_1_ and g_2_ were the peak values of the rate constants for CB detachment. The tilt of the myosin head relative to actin varied from 0 to h; *f*_1_ and *g*_1_ corresponded to a tilt equal from 0 to h, and *g*_2_ corresponded to a tilt >h; ϕ= (*f*_1_ + *g*_1_) h/2 = b. *N* was the cycling myosin CB number per mm^2^ at peak isometric tension; w was the maximum mechanical work of a single CB (w/e = 0.75) (Huxley, 1957); e was the free energy required to split one ATP molecule (Veech et al., 1979). According to Huxley's theory, only one ATP molecule was split per CB cycle. In vivo, the value of ΔG°′_ATP_ was nearby 60 kJ/mol. Thus, the value used for e was 10^−19^ J.

The molecular step size h was the translocation distance of the actin filament per ATP hydrolysis, produced by the swing of the myosin head. The parameter l was the distance between successive actin sites with which any myosin site could combine with actin. According to in vivo conditions (l>>h), the values of h and l were h = 10 nm and l = 28.6 nm (close to the semi‐helicoidal turn of the actin filament). Calculations of *f*_1_, *g*_1_, and *g*_2_ were based on the following equations (Lecarpentier et al., 1998):

(A3)
f1=Gg1

(A4)
g1=2wb/ehG

(A5)
g2=2Vmax/h

(A6)
$$\mathrm{po}=\left(w/l\right)\times \left[\left(f1\right)/\left(f1+g1\right)\right]$$

(A7)
$$\text{kcat}=\left(h/2\;l\right)\times \left[\left(f1g1\right)/\left(f1+g1\right)\right]$$

The number of active CBs (N) was equal to the ratio of the peak isometric tension and the elementary CB force (po); G was the curvature of the hyperbolic tension‐velocity relationship; Time stroke (ts) was the duration of the CB translocation (h); *V*_max_ was the maximum unloading muscle shortening velocity; muscle; kcat was the catalytic constant. The myosin concentration per g unit (MC) was calculated from Huxley's equations by dividing the CB number per g by the Avogadro number. The myosin weight per g was calculated knowing the myosin concentration (nmol/g of tissue) and the myosin molecular weight. Myosin (molecular weight ~528 kDa) is a hexameric protein consisting of two myosin heavy chains, two pairs of regulatory light chains, and two pairs of essential light chains (Jin et al., 2017). The myosin ATPase activity was the product of the myosin concentration and kcat. Thermodynamic force was the myosin concentration divided by the temperature (MC/T). Thermodynamic flow was the sliding velocity of myosin CB (v0 = h/ts). The rate of mechanical work (*W*_M_) was given by the equation: *W*_M_ = P_Hux_. V. At any given load level, the mechanical efficiency (Eff) of the uterine muscle was defined as the ratio of *W*_M_ and *E*_Hux_. Effmax was the peak value of Eff.

The rate of total energy release Eo _Hux_′ under isometric conditions was equal to a × b, the product of asymptotes which was proportional to the myosin concentration (MC) according to Eo′ = 8.48 MC. Thus, Eo was equal to 4.24 MC^2^. The thermodynamic force was Eo/T. The dimension of the thermodynamic force was an energy/T.
